# Single-Atom Sn-Loaded Exfoliated Layered Titanate Revealing Enhanced Photocatalytic Activity in Hydrogen Generation

**DOI:** 10.1021/acssuschemeng.2c06181

**Published:** 2023-02-15

**Authors:** Tuğçe Üstünel, Yusuke Ide, Sarp Kaya, Esmail Doustkhah

**Affiliations:** † Materials Science and Engineering, Koç University, 34450 Istanbul, Turkey; ‡ Koç University Tüpraş Energy Center (KUTEM), 34450 Istanbul, Turkey; § International Center for Materials Nanoarchitechtonics (WPI-MANA), National Institute for Materials Science, 1-1 Namiki, Tsukuba, Ibaraki 305-0044, Japan; ∥ Department of Chemistry and Life Science, Graduate School of Engineering Science, Yokohama National University, 79-5 Tokiwadai, Hodogaya-ku, Yokohama 240-8501, Japan; ⊥ Department of Chemistry, Koç University, 34450 Istanbul, Turkey

**Keywords:** Layered titanates, Hydrogen generation, Single atom Sn, Photocatalysis, Ammonia borane

## Abstract

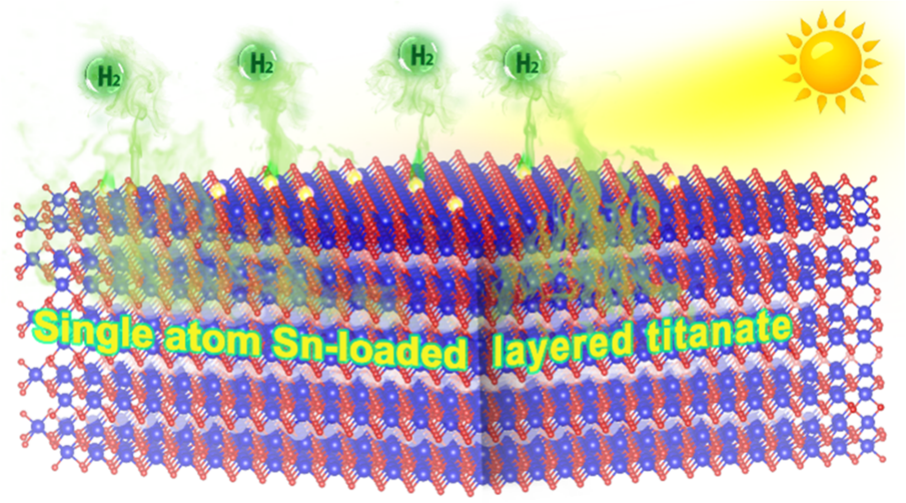

Green H_2_ generation through layered materials plays a significant role among a wide variety of materials owing to their high theoretical surface area and distinctive features in (photo)catalysis. Layered titanates (LTs) are a class of these materials, but they suffer from large bandgaps and a layers’ stacked form. We first address the successful exfoliation of bulk LT to exfoliated few-layer sheets via long-term dilute HCl treatment at room temperature without any organic exfoliating agents. Then, we demonstrate a substantial photocatalytic activity enhancement through the loading of Sn single atoms on exfoliated LTs (K_0.8_Ti_1.73_Li_0.27_O_4_). Comprehensive analysis, including time-resolved photoluminescence spectroscopy, revealed the modification of electronic and physical properties of the exfoliated layered titanate for better solar photocatalysis. Upon treating the exfoliated titanate in SnCl_2_ solution, a Sn single atom was successfully loaded on the exfoliated titanate, which was characterized by spectroscopic and microscopic techniques, including aberration-corrected transmission electron microscopy. The exfoliated titanate with an optimal Sn loading exhibited a good photocatalytic H_2_ evolution from water containing methanol and from ammonia borane (AB) dehydrogenation, which was not only enhanced from the pristine LT, but higher than conventional TiO_2_-based photocatalysts like Au-loaded P25.

## Abstract 2

Loading single atom Sn on protonated layered titanate revealing an enhanced photocatalytic green H_2_ generation through water splitting and ammonia borane dehydrogenation.

## Introduction

Replacing fossil fuels with green hydrogen (H_2_) has been accelerated in the current decade as one of the indispensable decarbonizing alternatives to mitigate CO_2_ emissions and harness climate change and its further consequences.^1^ After photocatalytic water splitting,^2^ dehydrogenation of small molecules by photocatalysis has become one of the key approaches for green sustainable H_2_ production.^3,4^ The borane compounds (as a high-content H_2_ reservoir)^5−8^ are unique candidates for H_2_ generation through a proper (photo)catalyst. Among the (photo)catalysts, layered materials are front liners in the catalysis toward H_2_ production^8,9^ due to their large theoretical surface area^10^ and their game-changing chemistry (e.g., functional groups) and physics (e.g., electron–phonon interactions),^11^ originating from their anisotropic structures.^12,13^ However, layered materials are usually in 3D bulk structures and need to be exfoliated since in the pristine bulk form, the layers are stacked through weak interactions, and the interlayers are inactive.^12^

Layered titanates (LTs) are of the most widely investigated layered materials as photocatalysts,^14−18^ but scarcely show promising photocatalytic activity in H_2_ generation applications under solar light irradiation due to their intrinsically large bandgaps in addition to the blockade of interlayers for any mass diffusion.^16,19^ Therefore, exfoliating the bulk crystal structure of LTs is found an applicable method to release the surface of the layers and further modifying with methods such as heterostructuring^20−23^ or doping^17^ and single atom loading with a photoactive species to narrow the bandgap is inevitable for photocatalytic applications.

LTs consisting of TiO_6_-interconnected layers can be found in different chemical compositions in which there is a strong electrostatic interaction among the layers. These LTs usually accompany an alkali metal counterion and, consequently, distinctly differ in their features and activities,^18^ but their large bandgaps are a feature in common.^15,17^ However, exchanging these alkali metals with other species can generate a significant change in the physicochemical activity of LTs.^24−28^ Therefore, the investigations of the photocatalytic activities of cation-exchanged LTs may bring serendipity features that require to be undertaken.

The pioneering works around the exfoliation of LTs were first established by Sasaki et al. with organic ammoniums, where they reported the osmotic swelling and subsequent exfoliation.^29^ Since then, some efforts have been made to modify the exfoliating method, including those without organic exfoliating agents. However, the structural conversion of LTs into a more stable phase (e.g., rutile or anatase) under mild or strict conditions has been reported.^30,31^ Thus, the major challenge of LTs is their (simultaneous) exfoliation without crystal phase change and without using an organic agent.^32^ Also, in a few successful cases, LTs have been exfoliated by some organic species such as alkylammonium salts; the intercalation of such species in the interlayers of LTs^33^ causes exfoliation or a partial phase conversion^32^ and, thus, makes the LTs impure or costly.^17,32,33^ For this reason, the optical features, precisely the photocatalytic properties, of the exfoliated pure LTs are still underexplored.

Nowadays, loading a single atom on semiconductors plays an important role in improving photocatalytic activity.^34,35^ However, in the case of the LTs, single-atom loading can barely be found. One of the critical challenges in single-atom catalysis is to stabilize the chemical state of the single atom on a semiconductor through the adjacent heteroatoms. Moreover, the single-atom oxidation state alteration is another challenge that is drastically impacted by the crystal structure and chemical composition of the surface of the semiconductor.^36−38^

Herein, we investigate the exfoliation of LT in dilute HCl water without any organic species and the roles of Sn^2+^ loading on the exfoliated LT nanostructures (exf-HTO) in photocatalytic ammonia borane (AB) dehydrogenation and hydrogen evolution reaction (HER) from water at room temperature. We demonstrate the single atom Sn-loaded exf-HTO (Sn/exf-HTO), in an optimal ratio, shows considerably high photocatalytic activity than conventional TiO_2_ photocatalysts, including P25 and Au nanoparticle-loaded P25.

## Experimental Section

### Materials and Characterizations

All reagents and solvents were purchased commercially and used without further purification. TiO_2_ (P25; 97%), SnCl_2_·2H_2_O (99.8%), K_2_CO_3_ (>99.0%), Li_2_CO_3_ (>99.0%), HCl (>37%), gold(III) chloride trihydrate (>49%), C_2_H_5_OH, and NH_3_BH_3_ were purchased from Sigma-Aldrich. Ultrapure water (≥18.2 MΩ·cm) was used throughout the experiments. X-ray diffraction (XRD) patterns were collected by using a powder X-ray diffractometer (Bruker D2 Phaser X-ray Diffractometer) with Cu Kα radiation with a 10 kV beam voltage and a 30 mA current. UV–visible spectroscopy measurements were performed using a Shimadzu UV-3600 UV–vis-NIR spectrophotometer with the reflection mode. It was converted to the absorption spectra by Kubelka–Munk transformation. Raman spectroscopy measurements were done by using Renishaw inVia Raman Microscope with the 633 nm excitation laser source. X-ray photoelectron spectroscopy (XPS) measurements were conducted using a Thermo K-alpha spectrometer system with Al Kα radiation (*h*υ = 1486.7 eV) as the excitation source. Binding energies for the XPS spectra were corrected by setting C 1s binding energy to 284.5 eV. Photoluminescence (PL) emission and lifetime measurements were performed using an Edinburgh Instruments FLS1000 spectrometer. Scanning electron microscopy (SEM) images were recorded by a Zeiss Ultra Plus microscope with an accelerating potential of 20 kV. Transmission electron microscopy (TEM) images were obtained by a 200 kV aberration-corrected TEM/STEM Hitachi HF5000 equipment. Photocatalytic activity measurements were performed using an LCS-100 solar simulator with 1.0 SUN AM 1.5G output from a 100 W xenon lamp. The quantification of H_2_ was done by a gas chromatograph (7820A, GC-System, Agilent) equipped with a thermal conductivity detector (TCD) and flame ionization detector (FID).

### Synthesis of KTLO

Layered potassium lithium titanate (K_0.8_Ti_1.73_Li_0.27_O_4_ named KTLO) was prepared by solid-state reaction method using TiO_2_, K_2_CO_3_, and Li_2_CO_3_ with the molar ratio of (4.1:6.0:0.7).^39^ The starting chemicals and three drops of C_2_H_5_OH were ground in an agate mortar with a pestle for 2 h, and the powder mixture was calcined in a muffle furnace at 600 °C for 20 h. After the first calcination, the mixture was cooled to room temperature. The sample was mixed for another 2 h and placed in the muffle furnace for the second calcination at 600 °C for 20 h.

### Protonation and Exfoliation of KTLO

The obtained KTLO (0.5 g) from the previous step was dispersed in 0.1 M HCl solution (100 mL) and stirred at room temperature. For the protonated layered titanate (HTO), the solution was stirred for 1 day at 600 rpm and separated by centrifuge, washed three times with C_2_H_5_OH (30 mL), and dried at room temperature. For the exf-HTO, the dispersion was stirred at a lower speed (400 rpm) for 3 days, and then, the supernatant was separated by centrifugation, and a fresh solution of 0.1 M HCl (100 mL) was added to the centrifuged powder and restirred under the same conditions at room temperature for 11 days. Afterward, the dispersion was centrifuged and dried in a freeze-dryer for 24 h.

### Preparation of Sn/exf-HTO

Adsorption of Sn on exf-HTO was done by adding 10 mg of exf-HTO to the different concentrations (5, 10, 15, 20, 40, 50, 60 ppm) of 20 mL SnCl_2_.2H_2_O solution in a test tube and mixing the solution in the dark for 3 h and then under the illumination of a solar simulator for 1 h. As the exfoliated layers have a negative charge (compensated with H^+^), cationic species can bind the layers via the electrostatic self-assembly process. The mixture was centrifuged and dried in a vacuum oven for 24 h. After the photocatalytic performance tests were done, Sn/exf-HTO was recycled via centrifugation and drying in the vacuum oven for 24 h as the resulting Sn/exf-HTO-rec sample.

### Photocatalytic Activity Tests

For performing the photocatalytic H_2_ production reaction from ammonia borane (NH_3_BH_3_), powder samples of KTLO, HTO, exf-HTO, and Sn/exf-HTO were dispersed in 4.5 mL of distilled water in a Pyrex test tube purged with N_2_ for 30 min. Before sealing with a rubber septum properly, 0.5 mL of 40 mM NH_3_BH_3_ was added. For the H_2_ evolution reaction from water, powder samples (5 mg) were dispersed separately in 5 mL of distilled water (20%V/V methanol) in a Pyrex test tube which was sealed properly and purged with N_2_ for 30 min. The test tube was illuminated via the solar simulator while stirring constantly. The amount of H_2_ produced was measured with the gas chromatograph, directly taking injections from the headspace of the Pyrex tube using a gastight syringe.

## Results and Discussion

**Scheme 1:**
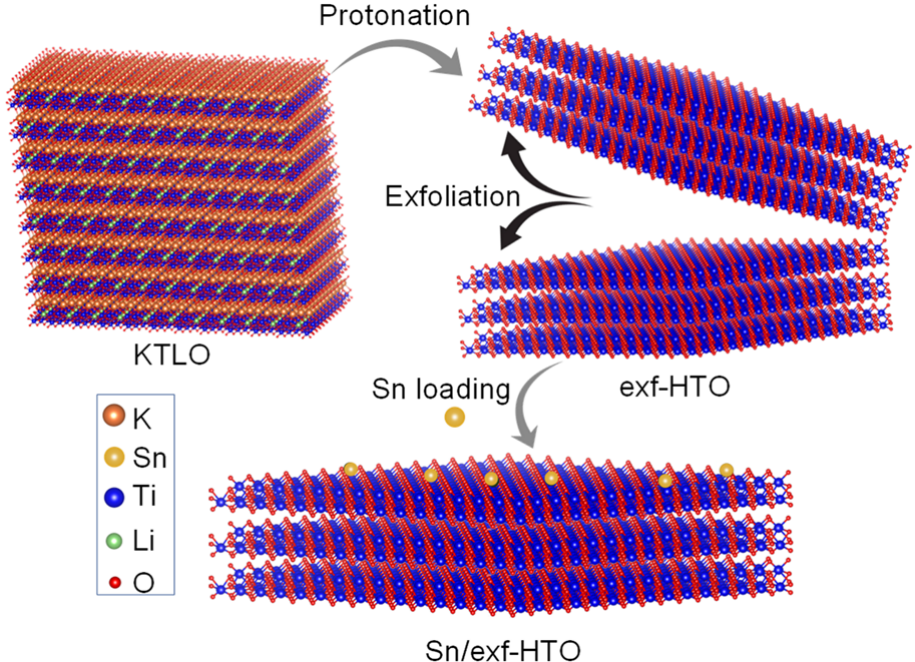
Schematics of Single Atom Sn Loading and Exfoliating KTLO into HTO

**Figure 1:**
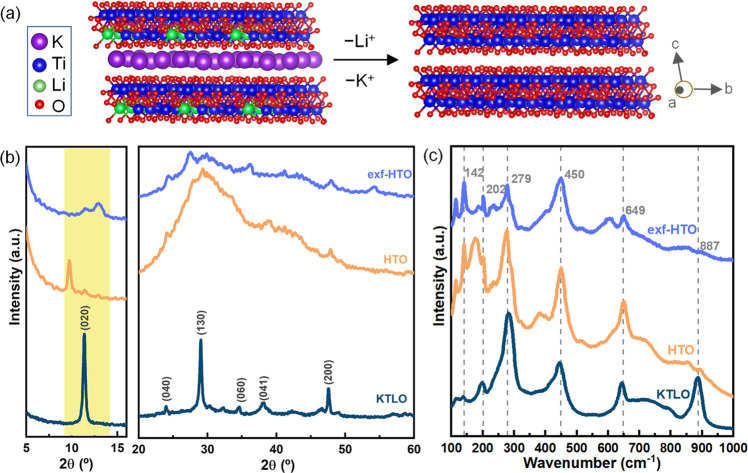
Crystal structures of KTLO and HTO from the edge view (020) plane. (a) XRD patterns (b) and Raman spectra (c) of KTLO, HTO, and exf-HTO.

Usually, the protonation, cation exchange, and exfoliation of LTs are three crucial types of postmodifications that can be found around developing LTs for various applications. Here, we present a new facile method for the exfoliation of LTs along protonation through long-term slow agitation of LT in a dilute HCl solution into a few-layered HTO. A step further, we succeeded in loading a single atom Sn and, subsequently, a schematic procedure for the preparation of the single atom Sn-loaded exf-HTO is presented in Scheme 1 and Figure 1a.

We confirmed the successful synthesis of layered titanate KTLO through the XRD pattern and used it for further protonation (HTO) and exfoliation (exf-HTO). In the case of KTLO, a peak at 11.4° corresponds to the (020) plane with a *d* value of ∼0.8 nm, representing the interlayer space and the layer thickness (Figure 1b). A peak at 2θ = 29.1° also corresponds to the layered structure of the material, showing the (130) plane. Another characteristic peak at 47.4° corresponds to the crystalline lepidocrocite structure of layered titanate, showing the (200) plane. Once protonated, the first peak at a lower degree slightly shifts to a smaller angle (9.7° corresponds to a *d* value of ∼0.9 nm), showing that the interlayer space has been expanded, i.e., by intercalating water after exchanging the K^+^ and Li^+^ with H^+^, so that the *d* value undergoes a 0.1 nm expansion with respect to KTLO (Figure 1b). As confirmed before,^28^ the protonated LT usually exhibits broader peaks compared to the pristine KTLO. Once the exfoliation is performed, the first characteristic peak of the layered structure has slightly shifted to higher angles, with a significant decrease in the intensity compared to those for HTO and KTLO and has become broader, showing that the layers are remarkably exfoliated (Figure 1b). The peak at 29.1° after the treatment is also broadened, showing that the protonation and further exfoliation make the layers’ assembly disorder (exfoliated) with less long-range order to be detectable by XRD as a sharp peak (this justification will be proved by TEM observation in the later section). Moreover, the same peak at 29.1° has less intensity in exf-HTO than HTO, further confirming the exfoliation. A similar XRD reflection is also reported for an LT, which is exfoliated by a bulky organic amine.^40^

Raman spectra of the samples are shown in Figure 1c. Accordingly, the peak at 887 cm^–1^ indicates the distorted TiO_6_ with a short Ti–O bond,^41^ which may be more affected by the presence of K^+^/Li^+^, where after replacing it with H^+^, the relevant peak almost disappears. The peaks at 279, 450, and 649 cm^–1^ are assigned to Ti–O–Ti stretching modes of TiO_6_ octahedra units.^40^ In conclusion, by comparing the Raman spectra of KTLO with the HTO and exf-HTO, the only change that can be observed is related to the interlayer surface chemistry, emerging as a peak at 887 cm^–1^, while the main backbone has a similar structure.

**Figure 2:**
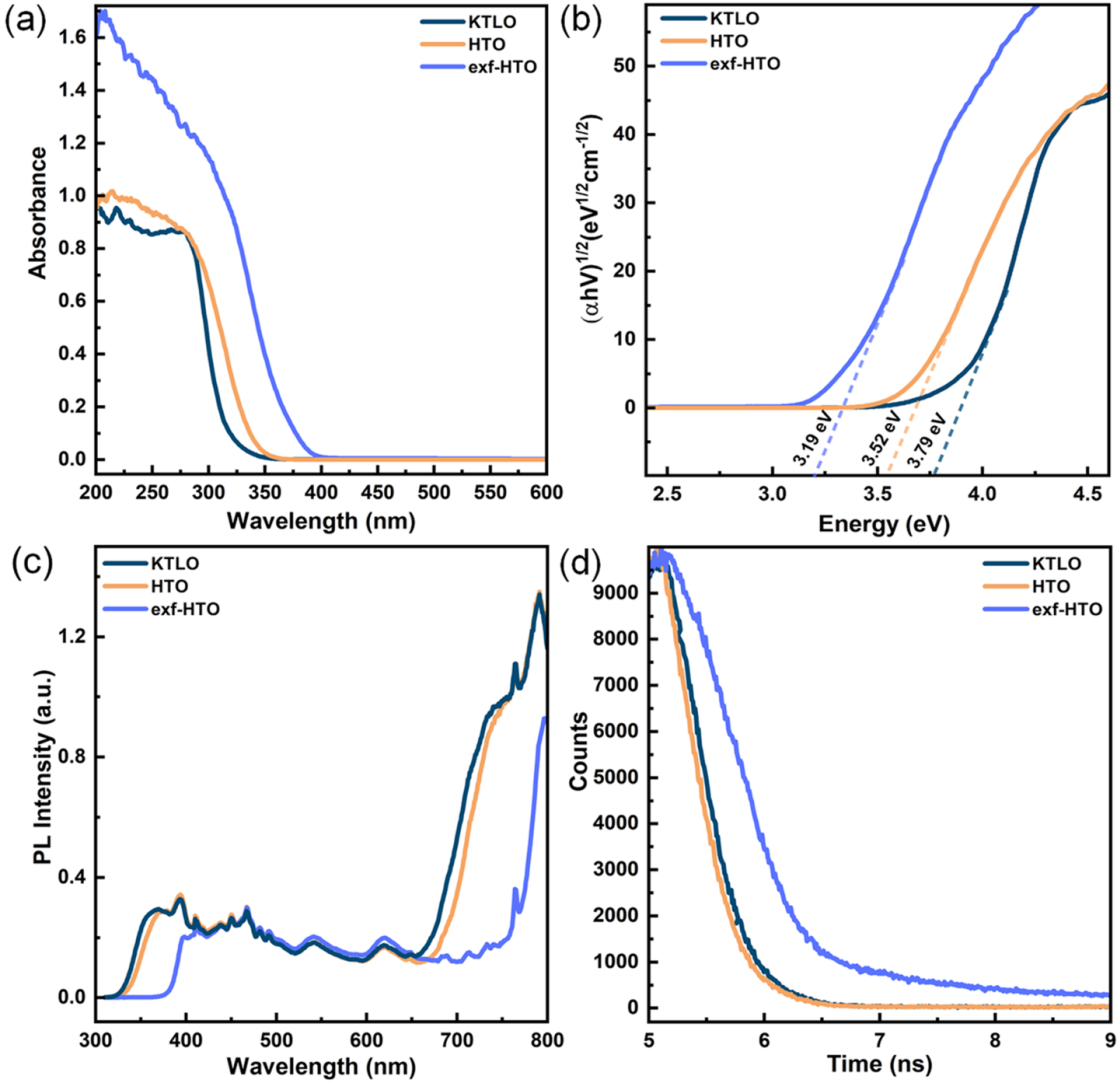
UV–vis spectra recorded in a diffuse reflectance mode (a), Tauc plot from UV–vis spectra using Kubelka–Munk conversion (b), emission spectra (c), and time-resolved PL decay profile (d) of KTLO, HTO, and exf-HTO.

UV–vis spectra and Tauc plots, which are used to determine light absorption properties and the bandgap of the photocatalysts, are presented in Figure 2a and b, respectively. While KTLO and HTO absorbed light with a wavelength shorter than 350 nm, the absorption property of exf-HTO reaches 400 nm. Tauc plots clearly demonstrated a change of the bandgap upon protonation and exfoliation, 3.19 eV was estimated to be the bandgap of exf-HTO while larger values, 3.52 and 3.79 eV were found for HTO and KTLO, respectively. A simple exfoliation method caused this remarkable change in the bandgap values and made this LT more sensitive to the light in the visible spectrum. The increased light absorption of the exf-HTO could be attributed to the introduction of new energy levels in the bandgap. Defects such as oxygen vacancies, titanium vacancies, and interstitials could be introduced on the surface or in the bulk during exfoliation, which could generate energy levels inside the bandgap.^24^

Exfoliation had a significant impact on the utilization of the charge carriers and their lifetime. It is seen in Figure 2c that pure KTLO and HTO exhibit a broad peak in the region of 330–400 nm, having a maxima at around 360 nm. The band edge emission and defect emission are two decay channels commonly suggested to describe the origin of the PL emission signals. Oxygen vacancy-mediated states resulting from the synthesis, protonation, and exfoliation could trap charge carriers and affect the emission profile. The PL spectra of exf-HTO show a red-shift of the peak maximum compared to that of KTLO and HTO, which can be attributed to the altered band structure, in agreement with its UV–vis absorption characteristics. Figure 2d demonstrates the fluorescence emission decays at a specific wavelength where the peak maximum is investigated to determine the lifetime of the charge carriers in samples. The recombination appears to be the slowest for the exf-HTO. The lifetimes were calculated by the deconvolution of the decay curves, and the values obtained for KTLO, HTO, and exf-HTO are 1.08, 0.94, and 1.57 ns, respectively. The increased lifetime of the charge carriers in the exf-HTOs reveals that the exfoliation-induced modifications are playing a critical role.

**Figure 3:**
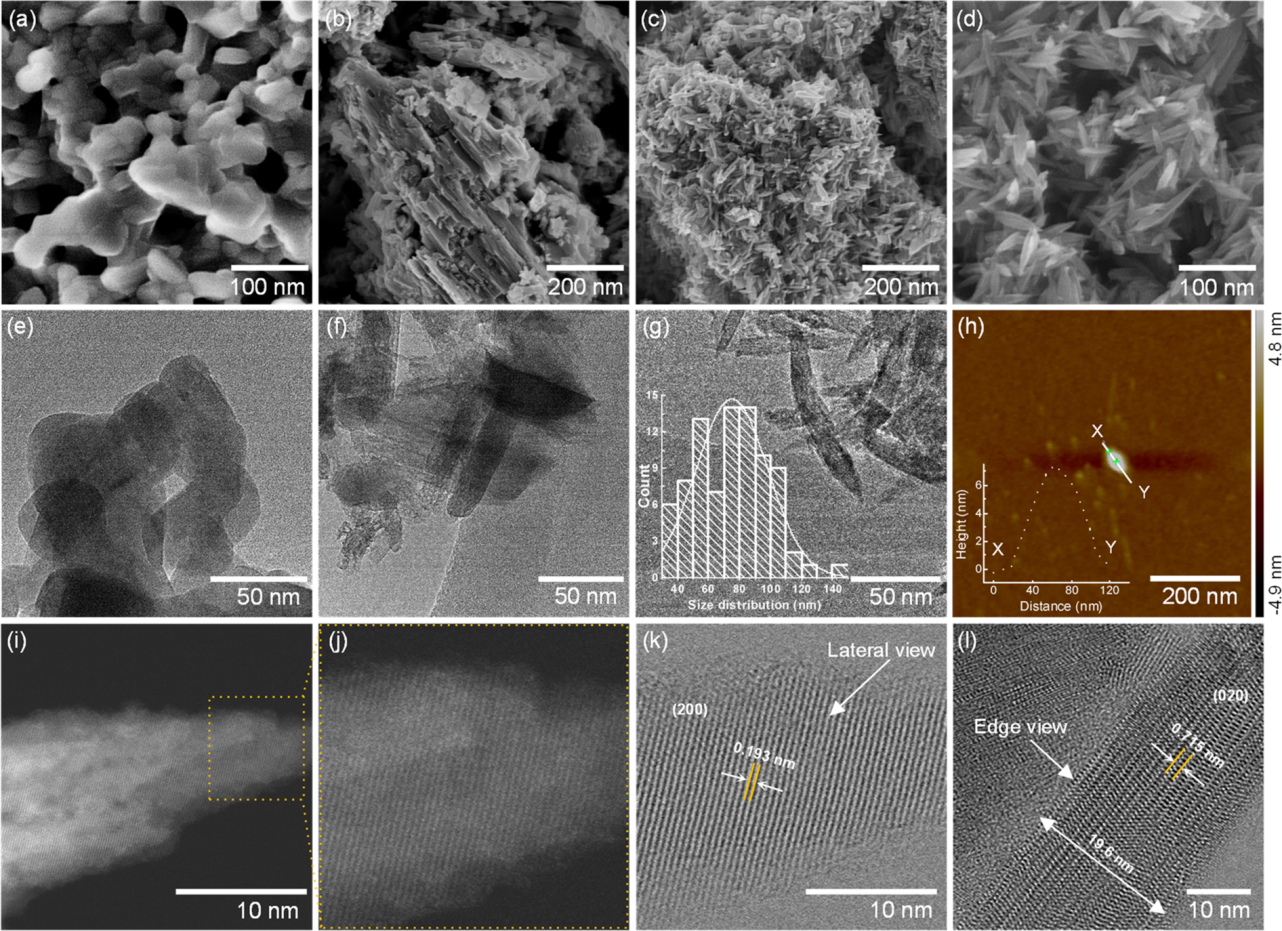
SEM images of KTLO (a), HTO (b), exf-HTO (c, d), and TEM images of KTLO (e), HTO (f), exf-HTO with a lateral width size distribution (g), AFM image of exf-HTO with a height profile (h), HR-TEM with low and high magnification (i, j), HR-HAADF-STEM with a lateral view and (200) planes according to the *d* value (0.193 nm) (k), and cross-sectional HRTEM of exf-HTO showing layer edges and 0.715 nm thickness (l).

We demonstrated the morphology and crystal structure and thickness of the exfoliated layered titanate through several microscopic techniques, including SEM, TEM, and AFM. The observed morphologies in SEM and TEM (Figure 3a,e) images match with each other and indicate a uniform nanostructure shaping from a randomly oriented morphology of KTLO (Figure 3a). Furthermore, by measuring the thickness of the representative exf-HTO particle, ∼8 nm, which can include the stacked from ∼11 layers of LT, where the lateral size shows ∼90 nm (Figure 3h). The observed lateral size in AFM is also a close value to the obtained average lateral size value from the SEM image of exf-HTO (∼75 nm, Figure 3g, see the inset). The thickness of exf-HTO compared to the normally protonated HTO (up to 35 nm, judging from the SEM image in Figure 3b) shows that our method significantly exfoliates the titanate layers along with protonation. The HR-TEM and (HR)-HAADF-STEM images of the exf-HTO show a single crystalline structure from the lateral dimension of the layer where the obtained d value from the observed facet is 0.715 nm, assignable to the (200) plane (Figure 3i–l). The edge view of exf-HTO also indicates a *d* value of 0.72 nm, which can be assigned to the obtained XRD peak at 12.3°, Figure 1b. Note that, due to the low long-range order of the exfoliated layers, the crystalline structure of the titanate layers can only be observed through the HR-HAADF-STEM and HR-TEM-captured images, where the XRD pattern is seldomly revealing this crystallinity due to the limited long-range order of the layers (Figure 1b). We also captured an HRTEM image from the edge view of the exf-HTO showing the stacking of 17 layers where the layers are free of K^+^ (Figure 3l), in comparison with a KTLO group (Figure S1); see the presence of K^+^ in the HR-TEM image that is captured in the edges view of the layers.

**Figure 4:**
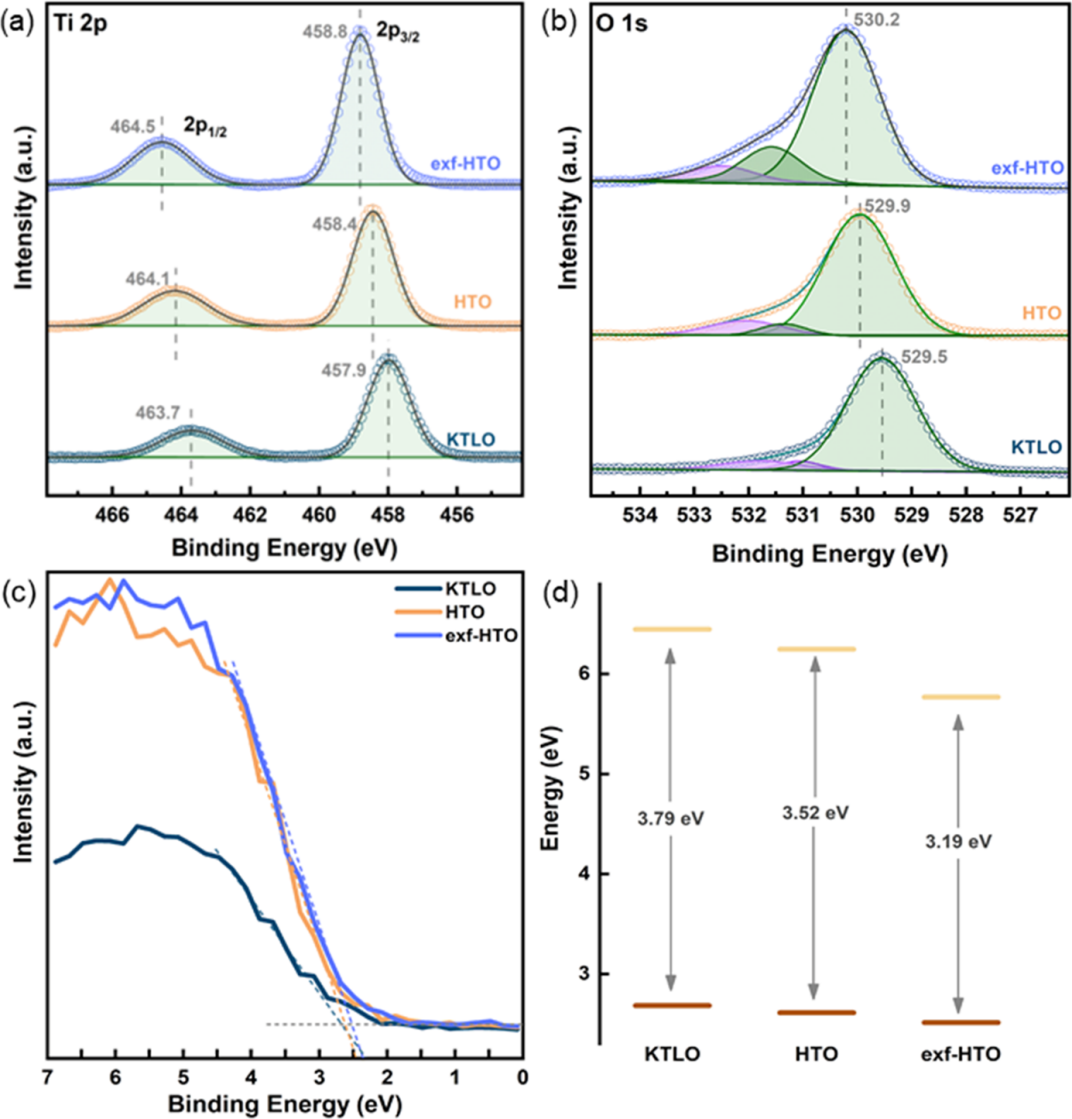
Ti 2p (a), O 1s (b), and VB (c) XPS spectra of KTLO, HTO, and exf-HTO, respectively. The alignment of the band edges based on VB edges determined by XPS and bandgap energies estimated by Tauc analysis (d).

XPS was used to investigate the surface composition and chemical state of photocatalysts. Ti 2p XPS spectra presented in Figure 4a exhibit peaks observed around 457 to 464 eV binding energies for Ti 2p_3/2_ and Ti 2p_1/2_, respectively. The Ti 2p_3/2_ peak shifted to higher binding energies from 457.9 eV (KTLO) to 458.8 eV (exf-HTO) as well as Ti 2p_1/2_ peak shifted from 463.7 eV (KTLO) to 464.5 eV (exf-HTO). HCl treatment results in K^+^ and Li^+^ ions at the interlayers of KTLO exchanged with H^+^. When protonation and, eventually, exfoliation occur, the bond distance between Ti and O gets shorter with the H^+^ ions presence since the electrostatic interplay between O and K^+^/Li^+^ ions is much stronger than that between O and H^+^ ions.^14^ The absence of the peaks in the high binding energy side of the spectra indicates that protonation and exfoliation treatments did not lead to the reduction of Ti. Similar shifts to high binding energies from KTLO to exf-HTO were also observed in O 1s spectra, as demonstrated in Figure 4b. O 1s spectra were deconvoluted into three peaks. The main component represents oxide ions (Ti–O–Ti), high binding energy shoulder within ∼531.1–532 eV, on the other hand, are assigned to hydroxyl groups (−OH) coordinated to the surfaces of the layers. Exfoliation treatment increases the number of −OH groups since more surface layers are susceptible to hydroxylation, and water adsorption is created (the component at the highest binding energy).

Valence band (VB) XPS spectra presented in Figure 4c could provide energy positions of the occupied energy levels. A close inspection of the band edge position reveals that the VB maximum moves down in energy upon exfoliation. This binding energy change matches with the energy shifts observed in core levels. A combination of bandgap energies estimated by Tauc analysis and VB maxima obtained in Figure 4c could be used to construct relative changes in the energy positions of KTLO, HTO, and exf-HTO. As summarized in Figure 4d, the gradual drop in the VB energy position and smaller bandgap energy significantly lowers the conduction band (CB) position.

**Figure 5:**
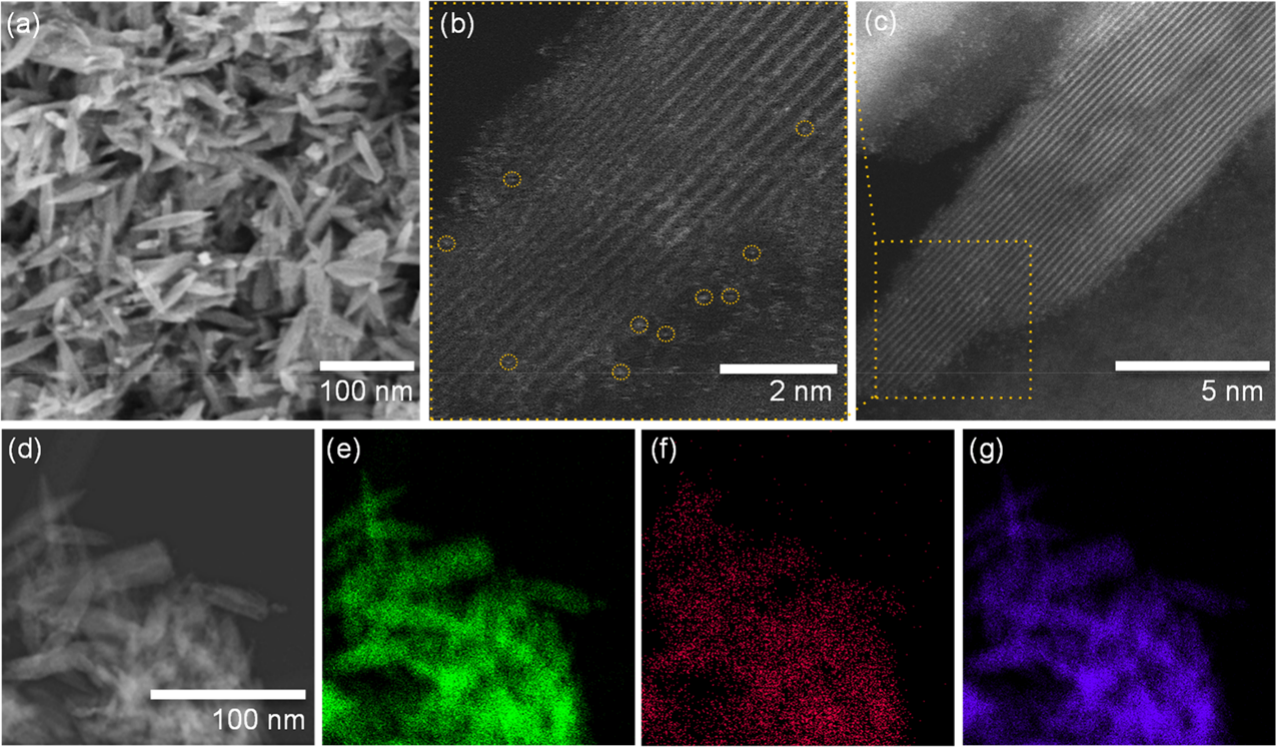
SEM image (a), TEM images (b, c), electron image (d), elemental mapping of O K_α1_ (e), Sn L_α1_ (f), and Ti K_α1_ (g) of Sn/exf-HTO.

We further explored the morphology of the exfoliated layered titanates after Sn loading and confirmed that the Sn loading process has no destructive effect on the nanostructure of the exf-HTO (see Figure 5a–c). The HR-STEM represents some light dots on the crystalline fringes of the LT, showing that the Sn species are in single-atom form and distributed on the surface. TEM-elemental mapping further confirms the thorough distribution of Sn uniformly along Ti and O on each particle (Figure 5e–g) without forming any Sn nanoparticles.

**Figure 6:**
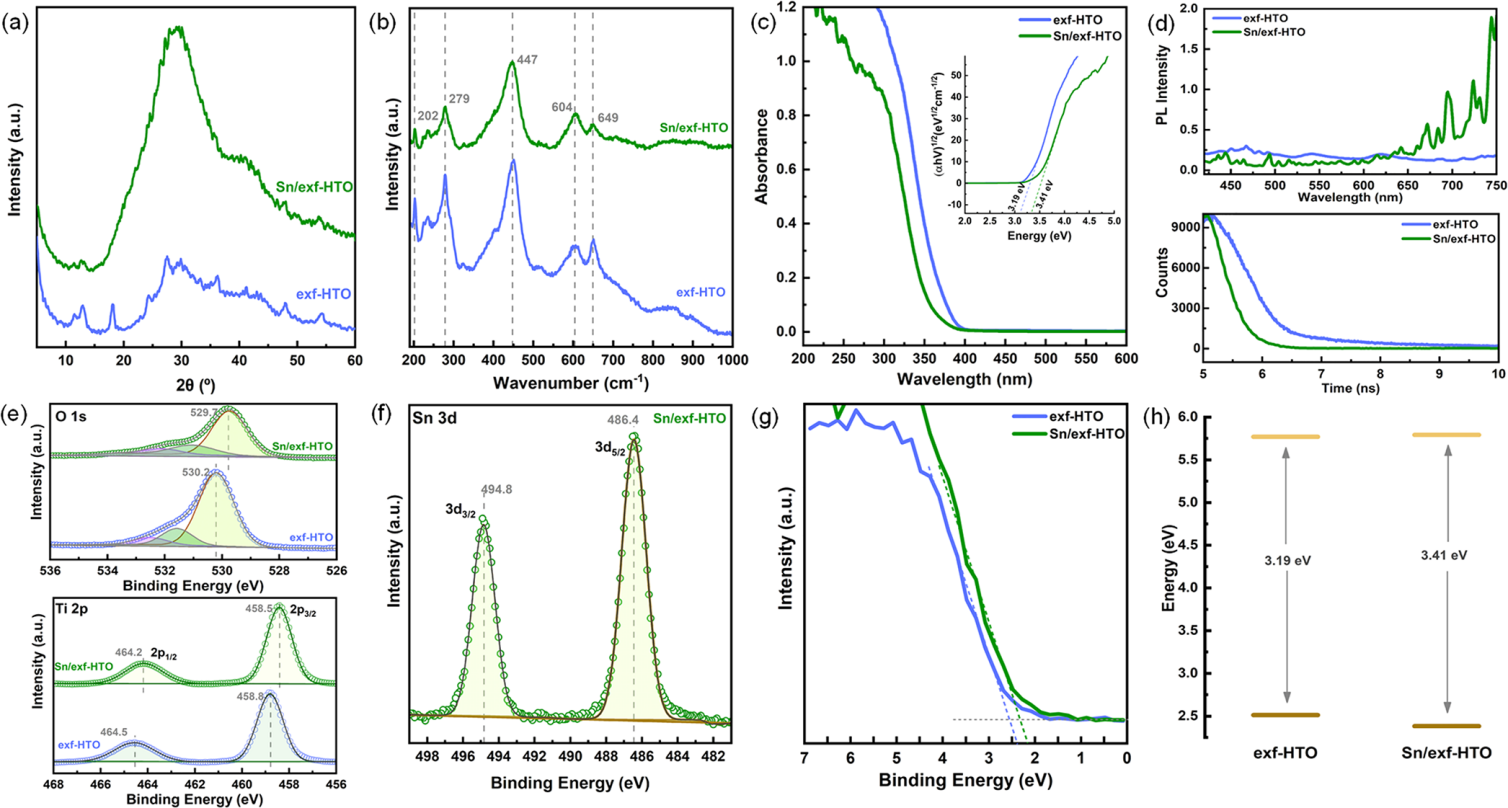
XRD pattern (a), Raman spectra (b), UV–vis spectra and Tauc plots (c), steady-state PL emission and time-resolved PL decay profile (d), Ti 2p-O 1s (e), Sn 3d (f), and VB (g) XPS spectra of exf-HTO and Sn/exf-HTO. The change in the alignment of the band edges is shown in (h).

Despite Sn single atoms being well distributed on the surface of LTs, the loading process has significantly changed the XRD pattern (Figure 6a). A broad background feature located around 30° similar to the one observed for HTO reappeared, and all the diffraction features remained buried under this background. No significant change in Raman spectral features has been observed (Figure 6b). This structural change could indicate that the long-range order has been lost upon Sn loading; however, the local structure and the coordination of the atoms remain intact. Sn single atoms not affecting vibrational features could be explained by their low loading. The reverse effect of the Sn loading is also evident in optical spectra. UV–vis spectra and respective Tauc analysis reveal band reopening, and bandgap energies shift from 3.19 to 3.41 eV (Figure 6c). Interestingly, steady-state PL intensity decreases within 400–600 nm (Figure 6d), suggesting that the recombination emission process has lost its dominance. Generally, the crystallinity and defects densities play a vital role in carrier extraction; however, the possible influence of the Sn loaded on the surface must undoubtedly be considered. Reduced PL intensity suggests that single-atom Sn layers could achieve better carrier extraction. Time-resolved PL decay measurements performed (Figure 6d) help us better understand the carrier decay behavior.^42^ Further decreased PL decay lifetimes from 1.57 to 0.97 ns point out that the efficiency of the charge carrier extraction could be increased by a single atom Sn layer.

Ti 2p and O 1s XPS main peak positions shifted to lower binding energies after Sn loading (Figure 6e). This binding energy shift seems to be related to the partial restacking of the layers as in the HTO structure. Sn single atoms remain on the surfaces of the layers. O 1s spectral deconvolution indicates that the peak previously assigned to H_2_O hydrogen bonded to surface OH groups have a lower intensity due to lowered exposed surface layers. Sn 3d XPS spectrum (Figure 6f) contains 3d_5/2_ and 3d_3/2_ spin–orbit components located at 486.4 and 494.8 eV, respectively, suggesting that Sn exists with the 2+ state on the surface.^43^ Comparing the Sn 3d XPS spectra of Sn^4+^-loaded exf-HTO with the one prepared here from Sn^2+^ (see Figure S2) further proves that the oxidation state of Sn in Sn/exf-HTO is remained +2. Sn with metallic character is not expected, no Sn (nano)particles were found under TEM observation, and BE of metallic Sn is not overlapping with the BE of the existing loaded Sn in Sn/exf-HTO.^44^ Sn loading leads to a small shift in VB maxima (Figure 6g), which re-establishes the VB and CB edge positions (Figure 6f).

### Photocatalytic Tests

**Figure 7:**
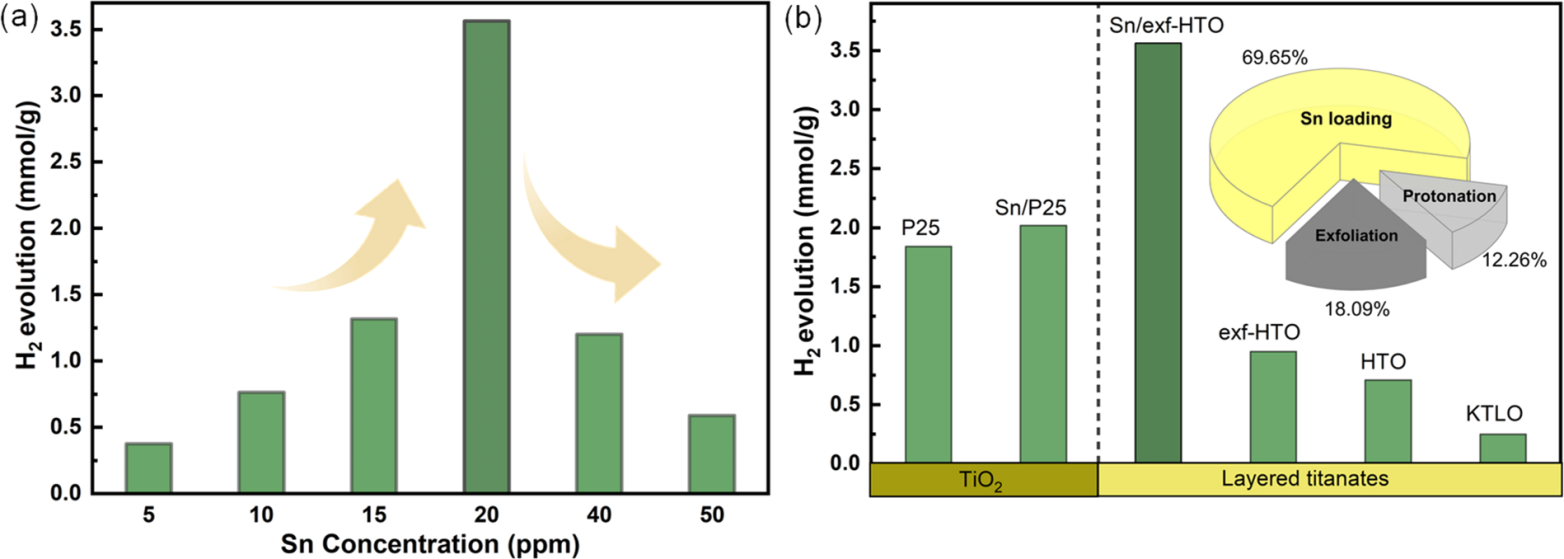
Solar-driven photocatalytic H_2_ evolution reaction from water containing methanol on Sn/exf-HTO loaded with different concentrations of Sn (a), photocatalytic activity comparison of Sn/exf-HTO (20 ppm) with other corresponding versions of LTs and the commercial TiO_2_ (P25) and Sn-loaded P25 (b).

Here, we tested the photocatalytic activity of the Sn-loaded protonated exfoliated HTO (Sn/exf-HTO) on the HER from water under solar light illumination. We also added MeOH (20 V/V%) to water for scavenging the photogenerated holes. First, the concentration of the treating solution of Sn for loading on exf-HTO was investigated. Accordingly, we treated the exf-HTO with different concentrations of Sn and found out that when the Sn solution is 20 ppm, the photocatalytic activity is in optimal activity compared to other selected concentrations, including 10, 40, and 50 ppm. The Sn-loaded exf-HTO obtained by treating it with 20 ppm of SnCl_2_ solution produces ∼3.5 mmol·g^–1^ H_2_ in 30 min of the illumination while for the other concentrations, we observed lower values (Figure 7a). Loading lower than 20 ppm of Sn is expected to have lower photocatalytic activities, while for the higher concentrations (e.g., 50 ppm) it was surprising, and we investigated the reason for the decrease in photocatalytic activity by studying its structure and morphology with SEM, TEM, and HAADF-STEM, see Figure S3. According to our observation, the layers aggregate, although the Sn concentration seems higher under HAADF-STEM, which points out that the Sn is still in a single atom form but causes the aggregation of the particles and a blockade of the direct light exposure to the active surface.

We further compared the photocatalytic activity of the other titanates and a TiO_2_ species with Sn/exf-HTO, in which Sn/exf-HTO has higher photocatalytic activity in H_2_ evolution (3.56 mmol/g) under identical conditions. For proving that the exfoliation step has a significant impact on the photocatalytic H_2_ evolution reaction, we compared exf-HTO with its corresponding HTO and found that the exf-HTO produces ∼241 μmol g^–1^ more H_2_ than the HTO in 30 min under the identical conditions. As a reference, we also evaluated the photocatalytic activity of KTLO to show that its activity is minor (Figure 7b) compared to the Sn/exf-HTO and the protonated ones. This low activity can be due to the fact that KTLO is still bulk layered material, and its activity is considerably poorer. Furthermore, along with the photocatalytic activity enhancement originating from protonation and exfoliation of the LT, loading Sn single atoms play a significant role in the photocatalytic activity enhancement when we compare the KTLO, HTO, and exf-HTO with Sn/exf-HTO as a trajectory of postmodification (Figure 7b, see the inset). This improvement in the photocatalytic activity after Sn loading can be due to the faster charge carrier separation, as judged by the PL decay profiles comparison of Sn/exf-HTO and exf-HTO, see Figure 6d. To our surprise, the Sn/exf-HTO exhibits even a better solar-driven photocatalytic H_2_ evolution activity than the commercial TiO_2_ (P25). The Sn-loaded TiO_2_ (P25) under the identical preparation conditions (see Figure 7) has a minor impact in the activity. This observation shows that the Sn species loaded on TiO_2_ is not showing the same mechanism of charge carrier photogeneration that occurs in the exf-HTO. Comparing the photocatalytic activity of Sn/exf-HTO with the previous reports for LTs, also further proves the excellent photocatalytic activity improvement after Sn loading, see Table S1.^15,45−47^

**Figure 8:**
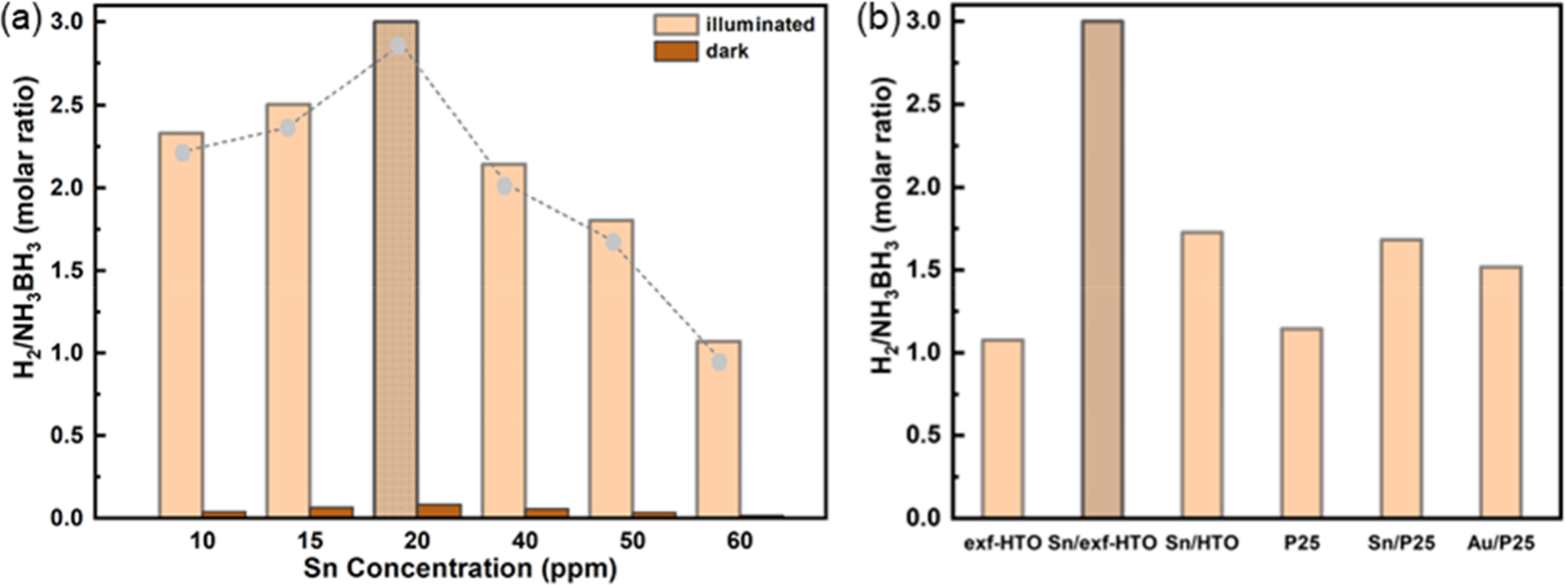
Comparison of Sn/exf-HTO photocatalytic activity in AB dehydrogenation reaction under illumination and dark conditions through different Sn loading amounts (a). Comparing AB dehydrogenation reaction rate of different TiO_2_-based photocatalysts with exf-HTO and Sn/exf-HTO (b).

We further evaluated the photocatalytic activity of the Sn/exf-HTO LT in AB dehydrogenation reaction at room temperature. AB can decompose into various forms, depending on the (photo)catalytic activity.^48^ However, each mole of AB usually converts to one mole of ammonium borate salt and three moles of H_2_ as follows:^48^
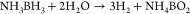
1We first investigated if Sn has any photocatalytic activity impact on AB dehydrogenation. Accordingly, when the reaction is carried out in the dark, the H_2_ generation is minimal, while in the presence of solar light, the AB dehydrogenation completes within 10 min. We also studied the impact of Sn loading amount on photocatalytic AB dehydrogenation and likewise hydrogen evolution reaction, the 20 ppm of SnCl_2_-treated exf-HTO exhibited a superior photocatalytic activity compared to selected lower and higher Sn concentrations treated with exf-HTO (Figure 8a). Here, the photocatalytic activity of Sn loading on exf-HTO was compared with the Sn-loaded P25 (a commercial TiO_2_), unexfoliated HTO, and bare HTO (Figure 8b). In all cases, the single atom Sn/exf-HTO revealed a superior activity than other Sn-loaded and unloaded titanates and TiO_2_. It is also important to note that the Au/P25 also showed less activity than the Sn-loaded exf-HTO, which can be pointed as an alternative to noble metals that are believed to be promising cocatalysts for TiO_2_-based materials.^49^ Moreover, Au as a noble metal cocatalyst, also possesses plasmonic activity that can enhance further but fails to show higher photocatalytic activity than the Sn-loaded exfoliated HTO. We also studied the photocatalytic activity of the Sn/exf-HTO in the dehydrogenation of the AB through illumination of monochromatized light to see the efficiency in the target photocatalytic reaction at various wavelengths. Accordingly, the photocatalytic activity of Sn/exf-HTO in the shorter wavelengths of UV range is significantly higher, a significant decrease in the photocatalytic activity occurs as it approaches to the visible range. This observation is matching well with the UV–vis absorption spectra, see Figure S4.

The photocatalytic activity of the recovered sample from AB dehydrogenation also indicates a slight decrease (Figure S5), confirming that the photocatalyst is reusable. Sn 3d XPS spectra of the recovered Sn/exf-HTO (Figure S6) after photocatalytic AB dehydrogenation was investigated to monitor any possible reduction of Sn species since AB can sometimes reduce the metals during the reaction.^50^ By comparing the Sn 3d XPS spectra of pristine and recovered Sn/exf-HTO, the oxidation state of the Sn had no sensible alteration during the photocatalytic AB dehydrogenation reaction. Furthermore, the XRD pattern and Raman spectral features of the Sn/exf-HTO-rec slightly differed from the Sn/exf-HTO, showing that the HTO structure is also sustainable and stable during the reaction process (Figure S7).

## Conclusion

In summary, we successfully exfoliated the layered titanate via a facile additive-free method, which provided an enhanced photocatalytic activity by narrowing the bandgap and prolonging the photogenerated electron/hole lifetime. Sn loaded on exf-HTO was found in single atom form homogeneously distributed on the surface and Sn seemed to be agglomerated when its loading was increased. A significant fraction of the enhancement of the photocatalytic activity toward hydrogen evolution and AB dehydrogenation reactions was due to the Sn single atom decoration on exf-HTOs. The oxidation state of Sn, the morphology, and the lepidocrocite structure of the exf-HTO were found to be sustainable during the photocatalytic reactions. Therefore, the single-atom Sn species can be proposed as an excellent candidate to trigger the photoactivity of the exfoliated layered titanates. At the same time, the other metals can be further explored from the perspective of the exfoliated layered titanates.

## Supporting Information Available

Preparation and XPS analysis of Sn^4+^-loaded exf-HTO, photocatalytic activity tests, and results at specific wavelengths, and XRD patterns, Raman spectra, XPS analysis, SEM, TEM, HAADF-STEM, and photocatalytic activity results for recycled Sn-loaded exf-HTO, the comparison table of photocatalytic activity (PDF)
