# Supplementary material for: Single-Atom Sn-Loaded Exfoliated Layered Titanate Revealing Enhanced Photocatalytic Activity in Hydrogen Generation
Source: ACS Sustain Chem Eng. 2023 Feb 15;11(8):3306–15. doi: 10.1021/acssuschemeng.2c06181 (PMC9976351; doi:10.1021/acssuschemeng.2c06181)
Supplement: Supplementary file 1 — sc2c06181_si_001.pdf [file sc2c06181_si_001.pdf]

Supporting Information

Single-atom Sn-loaded Exfoliated Layered Titanate Revealing an Enhanced Photocatalytic Activity in Hydrogen Generation

Tuğçe Üstünel,[1,2] Yusuke Ide,[3,4] Sarp Kaya,*[,1,2,5] Esmail Doustkhah*[,2]

[1] *Materials Science and Engineering, Koç University, 34450 Istanbul, Turkey*

[2] *Koç University Tüpraş Energy Center (KUTEM), 34450 Istanbul, Turkey*

[3] *International Center for Materials Nanoarchitechtonics (WPI-MANA), National Institute for Materials Science, 1-1 Namiki, Tsukuba, Ibaraki, 305-0044 Japan*

[4] *Department of Chemistry and Life Science, Graduate School of Engineering Science, Yokohama National University, 79-5 Tokiwadai, Hodogaya-ku, Yokohama 240-8501, Japan*

[5] *Department of Chemistry, Koç University, 34450 Istanbul, Turkey*

* Corresponding authors:

sarpkaya@ku.edu.tr (Sarp Kaya)

edoustkhahheragh@ku.edu.tr (Esmail Doustkhah)

Number of Pages: 6

Number of Figures: 7

Number of Tables: 1

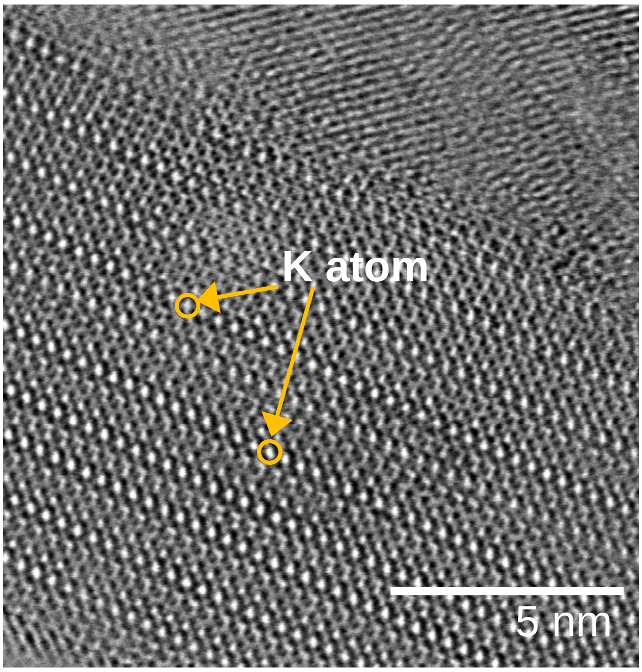

Figure S1. HRTEM image of K^+-unexchanged layered titanate (KTLO).

Preparation of Sn^{4+} loaded exf-HTO

Sn^{4+}-loaded exf-HTO was prepared by deposition of Sn^{4+} from 20 ppm of SnI_4 solution on exf-HTO. 10 mg of exf-HTO was dispersed in 20 mL of 20 ppm SnI_4 solution in a test tube, and the mixture was stirred at 400 ppm for three hours. After the solution was centrifuged at 3000 rpm for 20 minutes and washed with distilled water, it was put in a vacuum oven for 24 hours to dry at room temperature.

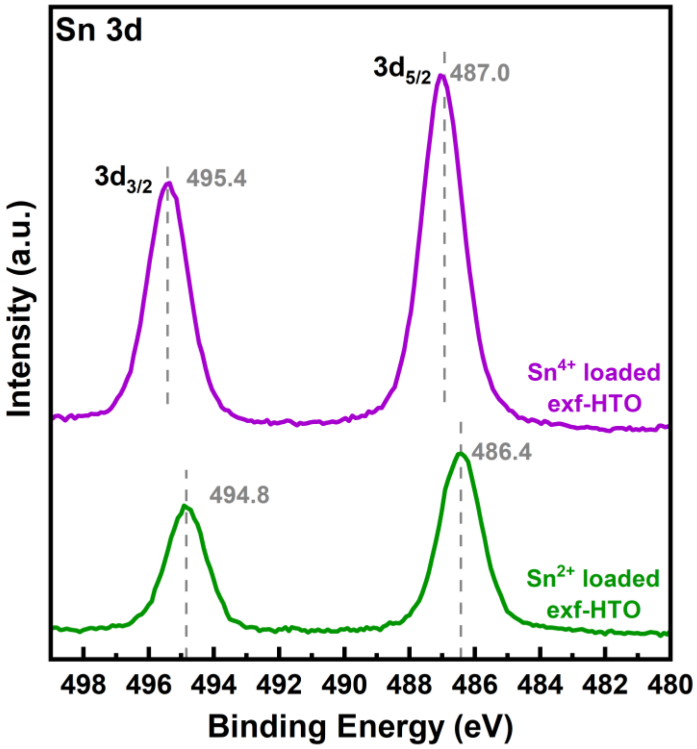

Figure S2. Sn 3d XPS spectra of Sn^{4+} and Sn^{2+} loaded on exf-HTO.

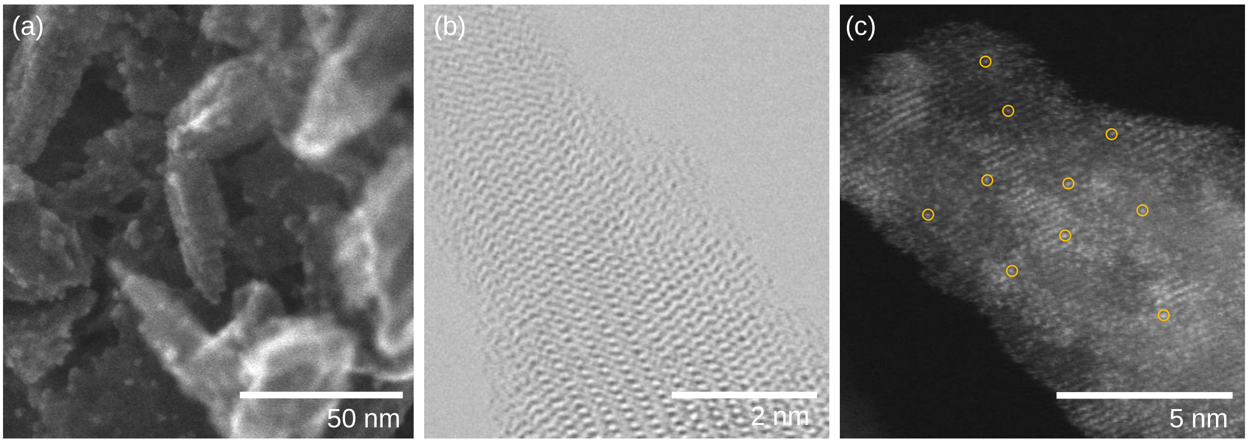

Figure S3. SEM (a), TEM (b), and HAADF-STEM (c) of Sn loaded exfoliated HTO (Sn/exf-HTO) with 50 ppm of Sn salt solution.

Photocatalytic activity tests at specific wavelengths

To make the photocatalytic H_2 evolution test by illuminating the light at specific wavelengths (320, 340, 360, and 380 nm), a Xe lamp (300 W) was used with a monochromator. The same procedure was followed to prepare the sample in a Pyrex test tube. After 5 mg of Sn/exf-HTO was dispersed in 4.5 mL distilled water and purged with N_2, 0.5 mL of 40mM AB was added and put under illumination for 30 minutes. A new sample was prepared at the same conditions

for each measurement of different wavelengths. The hydrogen amount was quantified with the gas chromatograph by taking injections.

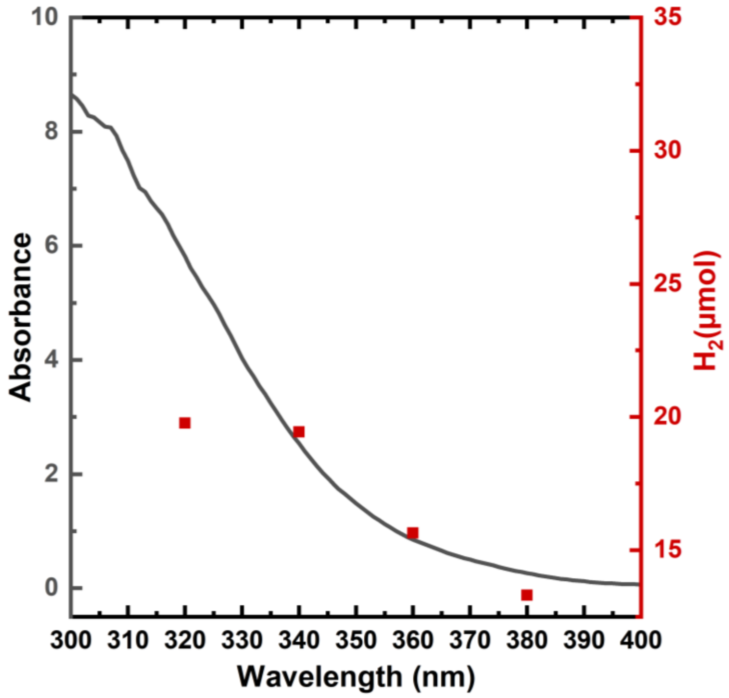

Figure S4. Hydrogen evolution from AB dehydrogenation with the absorbance at specific wavelengths.

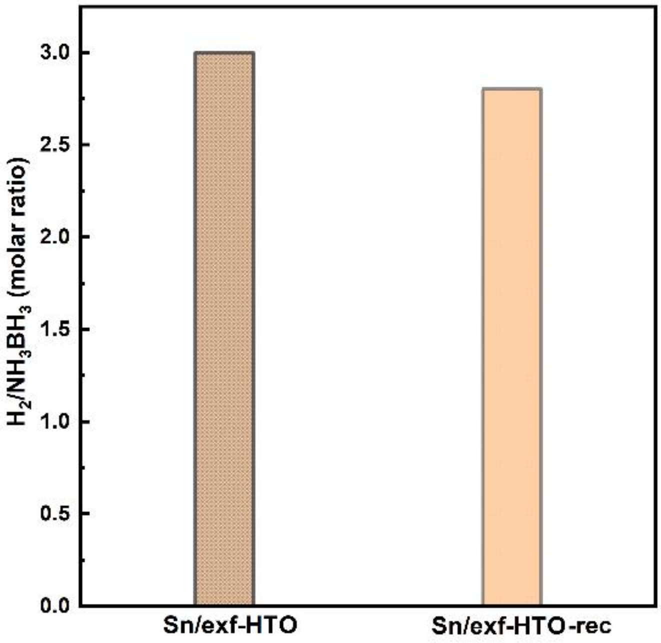

Figure S5. Photocatalytic activity of Sn loaded exfoliated HTO and the recovered Sn/exf-HTO in ammonia borane dehydrogenation.

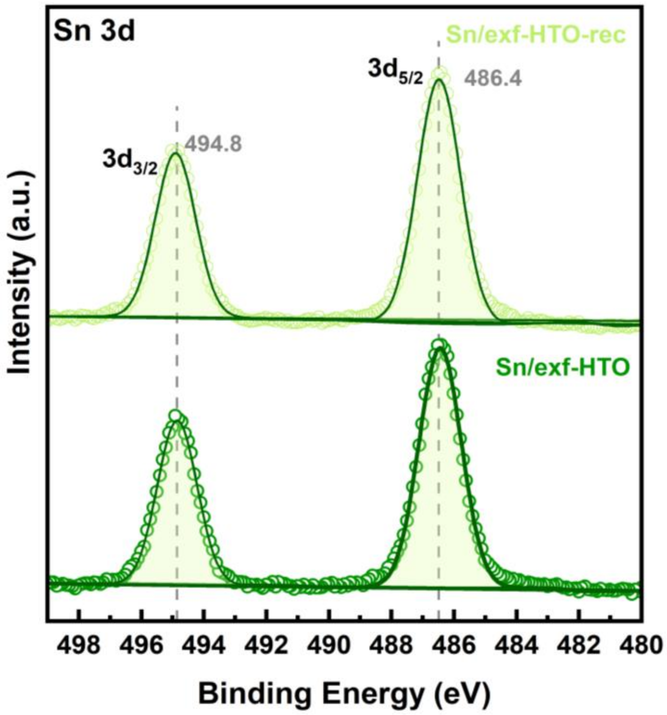

Figure S6. Sn 3d XPS spectra of Sn/exf-HTO and recovered Sn/exf-HTO-rec.

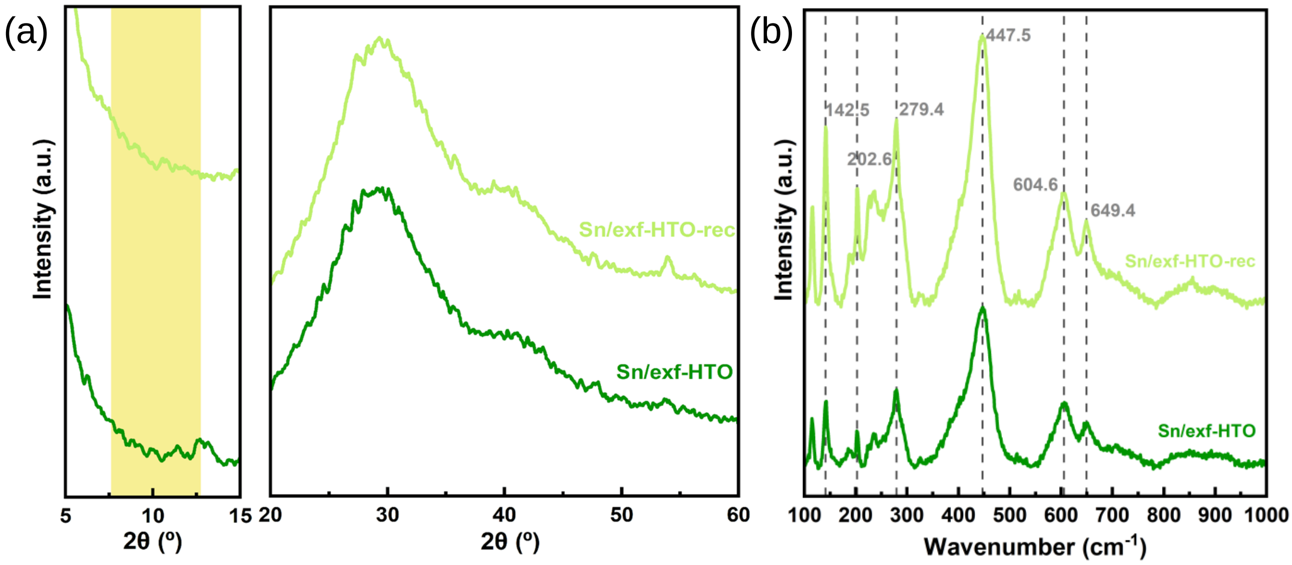

Figure S7. The XRD patterns (a) and Raman spectra (b) of Sn/exf-HTO and the recovered Sn/exf-HTO-rec.

Table S1. Comparison of photocatalytic activity of Sn/exf-HTO with previous reports which use layered titanates.

Catalyst	H_2 production rate mmol/g.h	Co-catalyst	Light wavelength range	Ref.
$Cs_{0.64}Ti_{1.79}Cu_{0.1}O_4$	5.1	Cu	UV-Vis	1
$H_2Ti_2O_5.H_2O$	9.28	Pt	300-1200 nm	2
$Na_2Ti_{3-x}Rh_xO_7$	1.97	Rh	>220 nm	3
$SrTiO_3$	0.202	Pt	320-780 nm	4
exfoliated HTO	14.2	Sn	UV-Vis	**This work**

References

(1) Pilarski, M.; Marschall, R.; Gross, S.; Wark, M. Layered cesium copper titanate for photocatalytic hydrogen production. *Applied Catalysis B: Environmental* **2018**, *227*, 349-355. DOI: https://doi.org/10.1016/j.apcatb.2018.01.039.

(2) Dong, F.; Zhang, G.; Guo, Y.; Zhu, B.; Huang, W.; Zhang, S. Flower-like hydrogen titanate nanosheets: preparation, characterization and their photocatalytic hydrogen production performance in the presence of Pt cocatalyst. *RSC Advances* **2020**, *10* (46), 27652-27661, 10.1039/D0RA03698F. DOI: 10.1039/D0RA03698F.

(3) Soontornchaiyakul, W.; Fujimura, T.; Yano, N.; Kataoka, Y.; Sasai, R. Photocatalytic Hydrogen Evolution over Exfoliated Rh-Doped Titanate Nanosheets. *ACS Omega* **2020**, *5* (17), 9929-9936. DOI: 10.1021/acsomega.0c00204.

(4) Kuang, Q.; Yang, S. Template Synthesis of Single-Crystal-Like Porous $SrTiO_3$ Nanocube Assemblies and Their Enhanced Photocatalytic Hydrogen Evolution. *ACS Applied Materials & Interfaces* **2013**, *5* (9), 3683-3690. DOI: 10.1021/am400254n.
